# Effect of the casein phosphopeptide-amorphous calcium phosphate fluoride (CPP-ACPF) and photobiomodulation (PBM) on dental hypersensitivity: A randomized controlled clinical trial

**DOI:** 10.1371/journal.pone.0225501

**Published:** 2019-12-02

**Authors:** Mariangela Ivette Guanipa Ortiz, Cristiane de Melo Alencar, Brennda Lucy Freitas De Paula, Eliane Bemerguy Alves, Jesuína Lamartine Nogueira Araújo, Cecy Martins Silva

**Affiliations:** School of Dentistry, Federal University of Pará, Belém, Pará, Brazil; National Taiwan University, school of dentistry, TAIWAN

## Abstract

**Objective:**

This randomized controlled clinical trial aimed to evaluate the effect of the casein phosphopeptide-amorphous calcium phosphate fluoride (CPP-ACPF) and photobiomodulation (PBM) in the treatment of dentin hypersensitivity (DH), and the impact of this on the health-related quality of life (HRQL).

**Methods:**

Eighty teeth with DH were randomized into four groups and received three treatment sessions: PLACEBO = placebo + LASER application mimicking; CPP-ACPF = CPP-ACPF + LASER application mimicking; PBM = placebo + LASER active application; CPP-ACPF+PBM = CPP-ACPF + LASER active application. Tactile (exploratory probe) and evaporative (triple syringe) stimuli were used to measure DH and were recorded with the aid of a visual analogue scale (VAS) after the 1^st^, 2^nd^ and 3^rd^ treatment sessions and one-month follow-up. The HRQL was recorded in the DH experience questionnaire (DHEQ).

**Results:**

The intragroup comparison showed a significant reduction in DH (p < 0.05) with both stimuli after one-month follow-up. The intergroup comparison with the evaporative stimulus showed that CPP-ACPF+PBM significantly reduced DH when compared to the rest of treatments, after one-month follow-up. CPP-ACPF+PBM group statistically differed from the other treatment groups in the DHEQ evaluation after one-month follow-up.

**Conclusion:**

After one-month follow-up, the association of CPP-ACPF with PBM was effective in the reduction of DH and promoted a positive impact on the HRQL of the participants of this study.

## Introduction

Dentin hypersensitivity (DH) is characterized by an acute and short-term pain, arising from vital dentin exposed to the oral medium, in response to thermal, evaporative, tactile, osmotic, or chemical stimulation [1,2]. Brännström’s hydrodynamic theory reports that DH pain is generated when the stimulus application, over the exposed dentin, changes the dentinal tubules fluid’s histophysiology. This rapid movement excites A-β and A-δ nerve fibers from the pulp’s periphery and transmits a signal that is perceived as pain [3,4].

DH prevalence ranges from 8% to 98% according to the evaluated population [5–7]. DH development is related to the loss of dental structure at the cemento-enamel junction and dentinal tubules exposure is caused by abfraction, abrasion, biocorrosion [6,8] associated or not with gingival recession [7,9].

Treatments that obliterate the dentinal tubules are ideal to reduce DH, however, their effectiveness will depend on their resistance to the various challenges of the buccal environment [10]. Recently, casein phosphopeptide-amorphous calcium phosphate fluoride (CPP-ACPF), a complex derived from the milk protein with fluoride addition has been introduced as a remineralizing agent [11]. CPP-ACPF can be useful in reducing DH by promoting the deposition of high concentrations of calcium, phosphate, and fluoride ions occluding the dentinal tubules [12].

Photobiomodulation (PBM) involves noninvasive application of red (600–700 nm) and near-infrared light (700–950 nm) [13], it is increasingly used in dentistry due to its analgesic, anti-inflammatory, and bio-stimulant effects. PBM increases the production of mitochondrial adenosine triphosphate (ATP) and the presence of β-endorphin suppressing P substance activity, it also decelerates the action potential velocity of C and Aδ fibers decreasing pain [14]. Also, PBM increases the odontoblasts tertiary dentin production obliterating dentinal tubules and reducing DH [15].

Several treatments for DH are available but none are considered the "gold standard," since most desensitizing agents have shown short-term effectiveness about up to two weeks from treatment conclusion [10,16], demonstrating the necessity of clinical trials to evaluate desensitizing treatments effectiveness in reducing DH and improving the health related quality of life (HRQL) of DH patients [17]. DH affects the patient’s psychosocial well-being by compromising daily activities such as brushing teeth, eating, drinking, and even social interaction [18].

CPP-ACPF and PBM association may generate a synergistic effect in DH treatment. This double-blind, parallel, placebo-controlled randomized clinical trial (RCT) evaluated the effect of CPP-ACPF associated with PBM on the permanent teeth of adult patients with DH. The null hypotheses of this study were: H0—There is no difference in the reduction in DH between the CPP-ACPF and PBM association and other types of treatments after one-month follow-up. H01—There is no difference in the improvement of HRQL among participants after treatment with CPP-ACPF associated with PBM and other types of treatment after one-month follow-up.

## Materials and methods

### Ethical aspects, sample size and study design

The CONSORT (Consolidated Standards of Reporting Trials) recommendations [19] and its extension, CONSORT- PRO (Patient Reported Outcomes), were followed (S1 Checklist) [20]. This RCT was approved by the health of science institute of ethics research committee of the University Federal of Pará (Approval number: 2.858.288 S1 Protocol) and was registered on the clinical trials registry (Registration number: NCT03750851; URL: https://clinicaltrials.gov/ct2/show/NCT03750851?cond=CPP-ACPF&rank=1). After being duly informed about the risks and methods of this study, all the participants signed an informed written consent in accordance with the Declaration of Helsinki [21].

The data obtained in a pilot study were introduced into the G Power^®^ program (Heinrich-Heine-Universität Düsseldorf, Germany), with a statistical power of 80%, a probability of error α of 5%, and effect size f estimative of 1.35 a total of 08 teeth per arm would be necessary. An inflation using the design effect was also calculated, based on an estimate ρ of 0.50, and assuming a 20% sample loss a total of 80 teeth were required. Participants were allocated into four groups according to the treatment applied as follows: PLACEBO: water-based placebo gel (K-Y^®^, Johnson & Johnson); CPP-ACPF: MI Paste Plus (Recaldent^™^, GC América); PBM: LASER—(Photon laser III, DMC U.S.A.); and CPP-ACPF+PBM: MI Paste Plus + LASER.

### Patient recruitment

Adults between 18 to 50 years of age, who reported dental sensitivity, were evaluated at the Federal University of Pará (UFPA), Brazil. Those who met the following inclusion criteria were recruited: at least two sensitive teeth with a response ≥ 4 on the 10 cm length visual analogue scale (VAS) (0—no pain and 10—unbearable pain) after tactile and evaporative stimulation and/or the presence of a non-carious lesion up to 2 mm deep [22], and/or class I gingival recession [23]. The exclusion criteria were: allergies to milk proteins; systemic diseases; carious lesions and/or pulpitis; presence of restorations on sensitive teeth; periodontal disease; cracks in the enamel; fixed orthodontic treatment; pregnant or nursing women; medication with analgesics or anti-inflammatories or have received desensitizing treatment during the three months prior to the recruitment date.

### Randomization and blinding

One of the research collaborators performed a block randomization using a numerical draw which allowed each participant to be allocated into one of the four groups under different arrangements (A4,1; A3,1; A2,1 and A1,1). The allocation concealment was maintained since the numerical draw was performed using numbered and coded papers. The code for each group was unknown by all: participants, principal investigator, clinical operator, and evaluator. In this double-blind study, participants were unaware of the desensitizing treatment received because both pastes were contained in similar recipients hindering visual identification and the texture, color and odor of the placebo was similar to MI Paste Plus. The evaluator was not aware of the group to which the participant belonged.

### Clinical protocol

All the participants received a toothbrush with soft bristles (Top Plus, Condor) and a dentifrice without a desensitizing action (EVEN Baby, Raymundo da Fonte) with oral hygiene instructions. Three desensitizing treatment sessions were performed with a 24-hour interval between each session.

The PLACEBO group received a water-based base paste application with a microbrush (Microbrush, 3M ESPE) on the cervical vestibular surfaces of each tooth for five minutes that was later rubbed for 20 seconds with a rubber cup (Prophy Cup, MICRODONT) coupled to a low speed handpiece (Contra angle 500, Kavo). Followed by PBM simulation, that was carried out by positioning the LASER tip at two points of each tooth, one at the center of the cervical region and another one in the middle third of the crown without light emission. The sound of the equipment was mimicked by an applicative on the cell-phone (Beep, Foncannon Inc).

CPP-ACPF group participants received the MI Paste Plus application following the PLACEBO group protocol, the PBM was simulated in the same way. For the PBM group, the placebo paste was applied as previously described and the LASER was applied using an infrared light spectrum with a wavelength of 808 nm, positioning its tip at the previously described points (60 J/cm^2^ at each point) for 16 seconds. In the CPP-ACPF+PBM group, MI Paste Plus and PBM were applied following the CPP-ACPF and PBM protocols.

DH evaluation was performed by requesting the participant to signal a compatible number to their pain sensation on the VAS after two stimuli: tactile was performed by sliding an exploratory probe in a cross-shaped way into the cervical region of the tooth. Evaporative consisted of the application of an air blast from a triple syringe for three seconds at a pressure of 40 psi (20 ± 3°C), perpendicularly to the buccal surface of the tooth (0.5 cm distance), the adjacent teeth were protected with cotton rolls [16,24]. DH was recorded in five moments: 1st registration: before starting the first treatment session; 2nd registration: after the first treatment session; 3rd registration: after the second treatment session (24 hours after the 1st treatment session); 4th register: after the third treatment session (24 hours after the 2nd treatment session); and 5th registration: after one month of follow-up.

### Dentin hypersensitivity experience questionnaire (DHEQ)

The participants’ self-reported evaluation was performed by giving them a questionnaire before the start and after one-month follow-up to determine the treatment impact on their HRQL [25,26]. Its summarized version consists of 15 questions that are answered according to a 7-point Likert scale. Higher scores indicate a poorer HRQL.

### Statistical analysis

VAS values were analyzed with the SPSS program (SPSS Statistics 25.0, IBM). A mixed-design ANOVA test was used to determine whether some of the treatments (between—subjects factor) were more effective for DH treatment throughout time (within—subject factor). Accounting for treatment effects at the different evaluation times were analyzed by a Tukey post-hoc test, tests of multiple comparisons were corrected using a Bonferroni adjustment. A 5% significance level was adopted for all the analyzes. Tooth level analyses were performed wherein each tooth individually contributed to the analyses. Hence this cluster data the subject was included as a random effect.

DHEQ data was analyzed by the Wilcoxon and Mann-Whitney tests. The impact of the effectiveness of DH treatment on the HRQL improvement was assessed by simple logistic regression. The difference between the final DHEQ and the initial DHEQ was transformed into binary data. Considering values ≥ 0 as worsening and < 0 values as an improvement in HRQL. The VAS final mean with an evaporative stimulus > 4 was considered as ineffective and ≤ 3 as effective in the DH treatment.

## Results

### Participants

**Fig 1:**
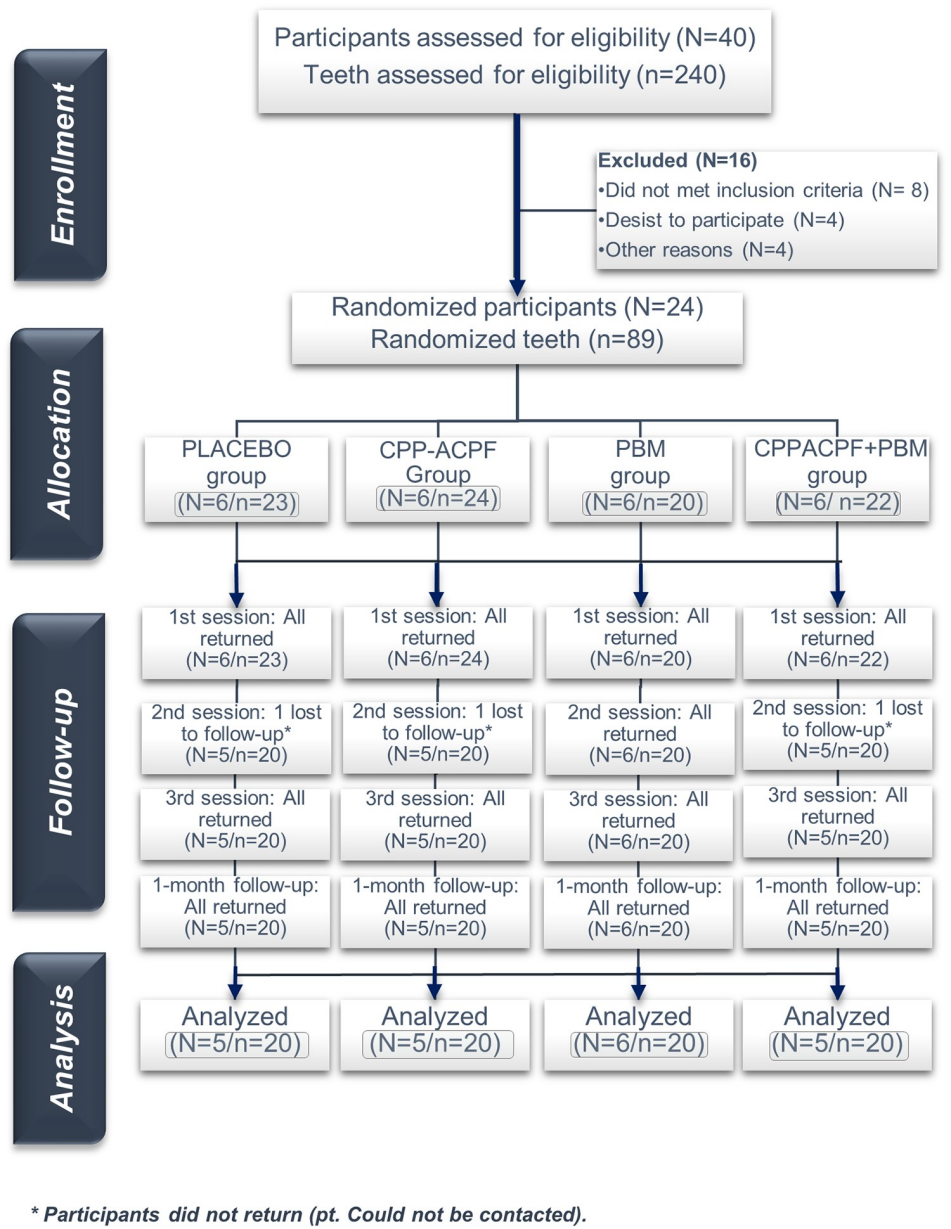
Flow diagram showing patient recruitment and follow-up. Adapted from the CONSORT flow diagram.

Between the 1^st^ June 2018 and the 20^th^ of October 2018, 40 participants were evaluated and 24 were randomized, treated, and accompanied according to Fig 1. Three participants (12.5%) did not return to the 2^nd^ session, 21 participants (87.5%) with 80 teeth completed all phases of the study.

### Demographic characteristics

Demographic characteristics of the 21 participants who completed the study are shown in Table 1. There was a higher proportion of female (N = 15; 71.43%) than male participants (N = 6, 28.57%). The mean age was 30 years (SD = 8.13). The most treated teeth were premolars (n = 51, 63.75%), followed by incisors (n = 20, 25%), canines (n = 7, 8.75%), and molars (n = 2, 2.5%). The analyses between the groups did not show any significant differences in these characteristics (p > 0.05).

**Table 1:** Participants demographic characteristics.

	PLACEBO	CPP-ACPF	PBM	CPP-ACPF+PBM	*p*
	(N = 5/n = 20)	(N = 5/n = 20)	(N = 5/n = 20)	(N = 6/n = 20)	
***Gender*,** ***N (%)***					
Female	3 (60)	4 (80)	3 (60)	5 (83.33)	0.793*
Male	2 (40)	1 (20)	2 (40)	1 (16.67)	
***Age*,** ***years***					
Mean	32.6	22.6	32.2	32.5	0.125**
Interval	22–47	18–27	23–38	26–50	
***Tooth type*,** ***n (%)***					
Incisors	5 (25)	5 (25)	4 (20)	6 (30)	0.300*†
Canines	2 (10)	4 (20)	0 (0)	1 (5)	
Premolars	11 (55)	11 (55)	16 (80)	13 (65)	
Molars	2 (10)	0 (0)	0 (0)	0 (0)	

* Fisher exact test;

** ANOVA test;

*^†^ Kruskal-Wallis test

### Tactile stimulus

A mixed ANOVA test demonstrated that there was a significant difference in the different evaluation times F = 44,661; (p < 0.001). While time and treatment interaction did not reflect a significant difference, F = 1,567; (p = 0.123). The mean and standard deviation of the VAS scores obtained with the tactile stimulus are described in Table 2. Using the baseline score as a reference, the intragroup analysis showed that in the PLACEBO and PBM groups the DH reduced after the 2^nd^ (p = 0.006), 3^rd^ session (p = 0.004), and One-month follow-up (p < 0.001). The CPP-ACPF and CPP-ACPF+PBM groups showed a significant reduction in DH after all sessions and one-month follow-up (p < 0.001).

**Table 2:** DH mean and standard deviation of the groups in the different evaluation times periods, using the tactile stimulus.

Groups (n = 80)	Baseline	1st session	2nd session	3rd session	One-month follow-up
	M (± SD)/ [% of DH reduction or increase on intragroup assessment]				
**PLACEBO** **(n = 20)**	5.65 (± 2.18) _**Aa**_ [–]	5.30 (± 2.89) _**Aa**_ [-6.19]	4.20 (± 2.75) _**Ba**_ [-25.66]	4.15 (± 2.70) _**Ba**_ [-26.54]	3.10 (± 2.63) _**Ba**_ [-45.13]
**CPP-ACPF** **(n = 20)**	6.20 (± 1.79) _**Aa**_ [–]	4.35 (± 2.28) _**Bab**_ [-29.84]	3.75 (± 2.34) _**Ba**_ [-39.52]	3.65 (± 2.50) _**Ba**_ [-41.13]	2.80 (± 2.97) _**Bab**_ [-54.84]
**PBM** **(n = 20)**	6.10 (± 2.17) _**Aa**_ [–]	6.10 (± 2.17) _**Aac**_ [0.00]	4.25 (± 2.97) _**Ba**_ [-30.33]	3.50 (± 2.65) _**Bab**_ [-42.62]	3.85 (± 2.48) _**Ba**_ [-36.89]
**CPP-ACPF** **+PBM** **(n = 20)**	5.35 (± 2.87) _**Aa**_ [–]	3.55 (± 3.68) _**Bb**_ [-33.64]	2.85 (± 2.81) _**Ba**_ [-46.73]	1.95 (± 2.58) _**Bb**_ [-63.55]	1.60 (± 1.79) _**Bb**_ [-70.09]

Different upper case letters represent significant intragroup statistical difference (p≤0.05); Different lowercase letters represent significant intergroup statistical difference (p≤0.05); p values calculated by the mixed ANOVA test and multiple comparisons tests with the Bonferroni post-hoc test

The intergroup comparison showed that the DH baseline mean was similar among all groups (p = 1.00). After the 1^st^ session, the CPP-ACPF+PBM group showed a higher and significant DH reduction when compared to the PLACEBO group (p = 0.053) and to the PBM group (p = 0.005). In the same period, the CPP-ACPF group showed a significant reduction in DH in relation to the PBM group (p = 0.05). After the 3^rd^ session, the CPP-ACPF+PBM group DH significantly reduced when compared to the PLACEBO (p = 0.009) and the CPP-ACPF groups (p = 0.043). After one-month follow-up, the CPP-ACPF+PBM group had a higher reduction in DH when compared to PLACEBO and PBM groups (p < 0.05) but did not differ from the CPP-ACPF group (p = 0.134).

### Evaporative stimulus

A mixed ANOVA test demonstrated that there was a significant difference among the evaluation times, F = 43.53; (p < 0.001), and in the time and treatment interaction, F = 2.68; (p = 0.003). The mean and standard deviation of the VAS scores obtained with the evaporative stimulus are described in Table 3. Using the baseline score as a reference, the intragroup analysis showed that all groups presented significant differences between the initial means and those obtained after one-month follow-up (p < 0.05). The PLACEBO group exhibited a significant reduction in DH after the 3^rd^ session (p = 0.024) and one-month follow-up (p = 0.004). The CPP-ACPF group showed a significant reduction after the 2^nd^ (p = 0.010), 3^rd^ session, and one-month follow-up, (p < 0.001). The PBM and CPP-ACPF+PBM groups presented a significant reduction in DH after the 1^st^ (p = 0.002), 2^nd^, 3^rd^ session, and one-month follow-up, (p < 0.001).

**Table 3:** DH mean and standard deviation of the groups in the different evaluation times periods, using the evaporative stimulus.

Treatment (n = 80)	Baseline	1st session	2nd session	3rd session	One-month follow-up
	M (± SD)/ [% of DH reduction or increase on intragroup assessment]				
**PLACEBO** (n = 20)	5.50 (± 2.35) ^**Aa**^ [–]	5.80 (± 2.95) ^**Aa**^ [+5.45]	4.55 (± 3.09) ^**Aa**^ [-17.27]	4.15 (± 3.17) ^**Ba**^ [-24.55]	3.60 (± 2.64) ^**Ba**^ [-34.55]
**CPP-ACPF** (n = 20)	5.75 (± 2.36) ^**Aa**^ [–]	4.50 (± 2.28) ^**Ba**^ [-21.74]	4.20 (± 2.31) ^**Ba**^ [-26.96]	3.45 (± 2.65) ^**Bab**^ [-40.00]	3.10 (± 2.69) ^**Ba**^ [-46.09]
**PBM** (n = 20)	6.40 (± 1.85) ^**Aa**^ [–]	4.55 (± 2.76) ^**Ba**^ [-28.91]	4.60 (± 2.37) ^**Ba**^ [-28.13]	3.60 (± 2.37) ^**Bab**^ [-43.75]	3.00 (± 1.84) ^**Ba**^ [-53.12]
**CPP-ACPF+** **PBM** (n = 20)	6.95 (± 2.74) ^**Aa**^ [–]	5.15 (± 3.42) ^**Ba**^ [-25.90]	4.55 (± 2.95) ^**Ba**^ [-34.53]	2.55 (± 1.99) ^**Bb**^ [-63.31]	1.60 (± 1.73) ^**Bb**^ [-76.98]

Different upper case letters represent significant intragroup statistical difference (p≤0.05); Different lowercase letters represent significant intergroup statistical difference (p≤0.05); p values calculated by the mixed ANOVA test and multiple comparisons tests with the Bonferroni post-hoc test.

The intergroup comparison showed a similarity in the VAS means obtained at the baseline evaluation and after the 1^st^ and 2^nd^ session among all the groups (p > 0.05). After the 3^rd^ session, the CPP-ACPF+PBM group had a significant reduction in DH when compared to the PLACEBO group (p = 0.050) but did not differ significantly from the other groups (p > 0.05). After one-month follow-up, the CPP-ACPF+PBM group showed a significant reduction in DH when compared to the PLACEBO (p = 0.007), CPP-ACPF (p = 0.040), and PBM (p = 0.050) groups.

### Dentin hypersensitivity experience questionnaire (DHEQ)

**Fig 2:**
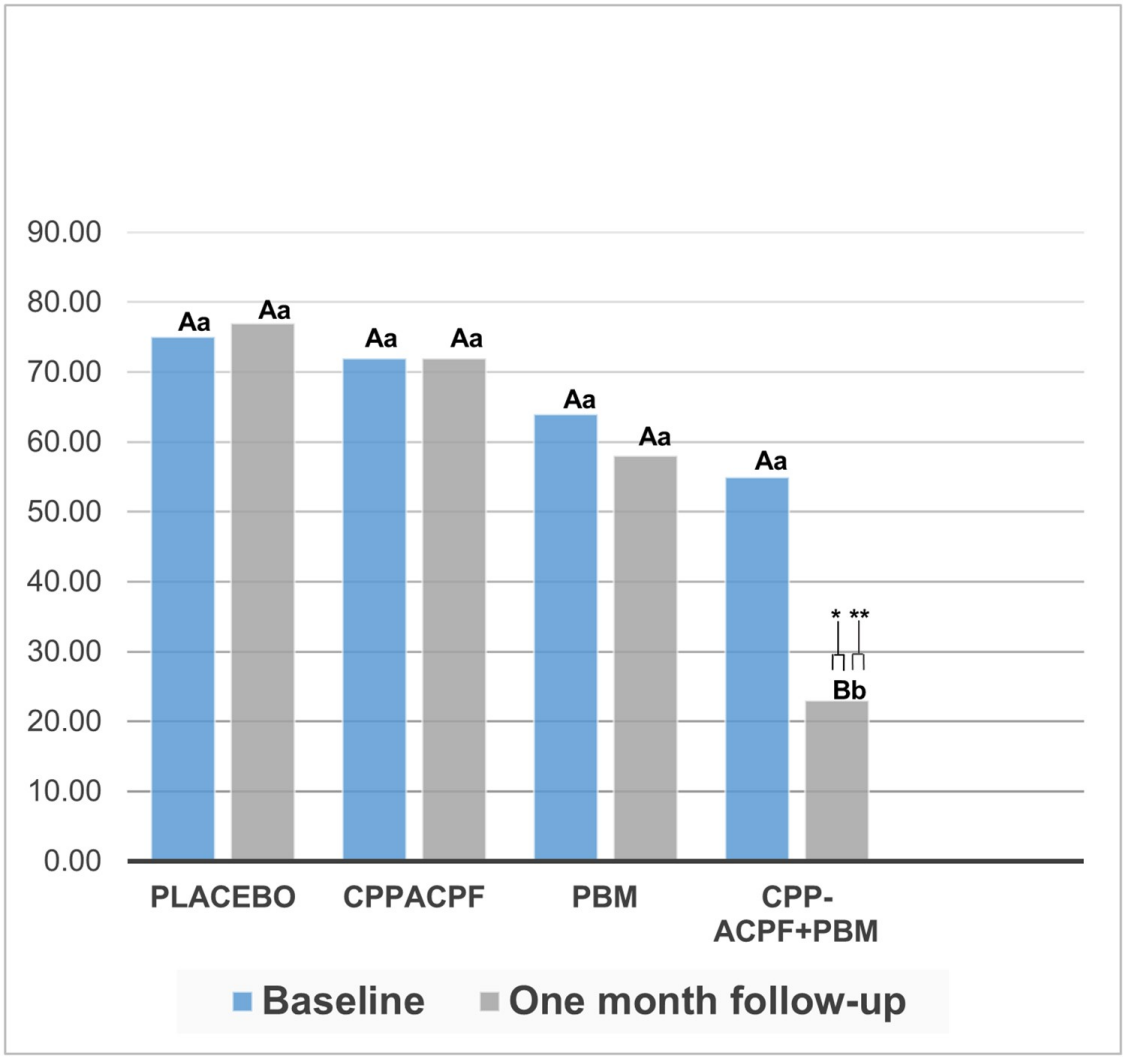
DHEQ results, median values before and after one-month follow-up of the desensitizing treatments application. * Different upper case letters represent significant intragroup statistical difference according to the Wilcoxon Test (p≤0.05); ** Different lowercase letters represent significant intergroup statistical difference according to the Mann-Whitney Test (p≤0.05).

The Wilcoxon test showed a significant difference in the overall analysis between pre-treatment versus post-treatment HRQL (p = 0.031). The intragroup analysis showed that the DHEQ median differences before and after the desensitizing treatment were not different in the PLACEBO (p = 0.893), CPP-ACPF (p = 0.345), and PBM (p = 0.500) groups. The CPP-ACPF+PBM group showed a significant improvement in the HRQL (p = 0.028). The intergroup analysis showed that the DHEQ medians at baseline were similar for all the groups (p > 0.05). After one-month follow-up, the CPP-ACPF+PBM group had lower DHEQ values, thus, a significant improvement in the HRQL when compared to the PLACEBO, Z = 2,739 (p = 0.006), CPP-ACPF and PBM groups, Z = 2.37 (p = 0.018) (Fig 2).

### HRQL and DH improvement

Of the 21 evaluated participants, 15 experienced an improvement in their HRQL. The DH treatment was effective in 12 participants. A simple logistic regression analysis showed a relationship of dependence between the HRQL improvement and the desensitizing treatment effectiveness after one-month follow-up. A desensitizing effective treatment has a chance of 91.67%, an OR = 13.7500; and a 95% CI = 1.21–156.66, (p = 0.0347) of improving the HRQL.

## Discussion

DH is related to repeated episodes of acute pain and a short duration, characterizing a chronic pain process [2]. The ideal desensitizing agent should have the following characteristics: be biocompatible, easy to apply, be painless, not changing the dental color, fast onset, and maintain a long-term effect [27]. In the RCTs DH is usually measured through the application of clinical stimuli and questionnaires are used to record the biopsychosocial impact of DH treatment on the patients’ quality of life [25].

The demographic analysis showed that most of the participants were female, agreeing with the previous studies (62% -72.2%) [28–30]. This is due to the greater frequency of dental brushing and/or consumption of acid diet foods, combining abrasive and erosive factors [17]. The sample mean age was 30 years, this is probably related to the fact that over the years the continuous deposition of tertiary dentin results in sclerosis of the dentinal tubules and in some cases pulp atrophy [17,31]. Altogether this fact decreases DH overtime and justifies the fewer elderly participants in the current study. Demographic characteristics were similar in all groups, indicating a strict selection criteria application, increasing the probability of finding a real association between desensitizing treatment and a reduction in DH [32].

The positive interaction between the time and treatment factors demonstrated the impact of the number of applications to achieve a better efficacy. After one-month follow-up, all treatments significantly reduced DH, including the placebo group, revealing effectiveness. The positive response to the placebo treatment may be related to the impact of psychosomatic phenomena, spontaneous improvement, positive responses by courtesy and experimental subordination [33]. The psychological impact is greater with the placebo effect obtained with the PBM simulation, since the participants believed they were receiving a high technology treatment compromising their pain perception [34].

The PLACEBO and the CPP-ACPF and PBM groups similar results could be explained by the fact that a positive professional approach has been related to DH reduction by increasing the participants trust and inhibiting the pain controlled by the central nerve system [27]. The placebo (a positive response based on the intervention rather than from an active ingredient) and Hawthorne (a change in subject behavior, e.g. tooth brushing, as result of participating in an observed study) effects could explained the overall DH reduction, also, this last effect could have reduced the impact of erosive/abrasive habits on DH [35]. Even though the associated group (CPP-ACPF+PBM) may have experience something similar, the overwhelming effect of the combined actives agents demonstrated a greater impact in the one-month follow-up result [36].

Casein phosphopeptide (CPP) is a casein-derived phosphoprotein whose phosphoserine sequences have the ability to bind and stabilize the soluble amorphous calcium phosphate (ACP). In the presence of saliva, ACP dissociates into calcium and phosphate which bind to the tooth surface and provide a reservoir of large amounts of calcium and phosphate ions, maintaining a relative state of dental mineral supersaturation and favoring remineralization by precipitating in the form of hydroxyapatite (HA), occluding the dentinal tubules [12,37]. Fluoride (900 ppm) was introduced in this formulation (CPP-ACPF), favoring tissue remineralization. CPP-ACPF showed effectiveness in the reduction of DH after one-month follow-up, this can be attributed to the increase in bioavailable calcium and phosphate concentration and to the synergistic effect of the fluoride of its formulation with these ions, incorporating them into the dental biofilm forming mineral precipitates which are stable and contain fluorapatite (FA) and establish a wide remineralizing network [11,38,39].

In the PBM group with the evaporative stimulus, a significant reduction in DH was evidenced after the 1st session, showing the immediate analgesic action produced by the PBM. DH treatment with low energy dose emissions stimulate circulation, suppress neuronal transmission at the dentin-pulp interface when inhibiting the depolarization by formation of temporal bends’ in the C fibers axons, blocking 30% of neuronal activity after 10–20 minutes of application [40]. PBM’s ability to stimulate normal physiological functions and stem cell differentiation through reactive oxygen species and the generation of growth factors [41], eliminates pulpal damage and increases cellular activity of the odontoblasts, stimulating the deposition of tertiary dentin and subsequent tubular obliteration, reaching a lasting and stable effect, justifying the reduction in DH after one-month follow-up [34,40].

The adhesion of the CPP-ACPF complexes to the dentinal surface relies on the higher ability of the CPP-ACP compounds to dissolve in aqueous solutions, such a saliva, which generates a diffusion gradient [42], that combined to the adhesive nature of the CPP allows the CPP-ACPF nanocomplexes to locate over the supragingival plaque and the dentinal surface [37,43]. In this scenario, the negatively charged residues of the CPP are attracted to the exposed positively charged apatite crystals faces on the surface allowing the CPP adsorption and the release of the cargo of calcium, phosphate and fluoride ions contained within the cross-linked nanocomplexes [38,44,45]. The release of these ions onto the dentinal surface has a remineralizating effect through the precipitation as fluorapatite crystals forming a nanofilament coating, that occludes the open dentinal tubules. [45,46]

Few studies evaluate desensitizing products based on calcium and phosphate [47]. No studies assess the CPP-ACPF+PBM association in the DH treatment, hindering the results comparison with the literature [27]. CPP-ACPF and PBM association showed the largest reduction in DH after one-month follow-up when compared to the other treatments, rejecting H0. In accordance to a recent *in vitro* study [48] the synergistic effect can be explained as follows: consecutive clinical sessions of PBM may have favored a better adhesion of CPP-ACPF, as a consequence of the dentin surface modification induced by the PBM, which maintained the phosphate, calcium and fluoride previously deposited by CPP-ACPF over the dentinal surface, leading to the formation of a more stable and acid-resistant mineral precipitate on the dental surface, favoring an obstruction of the dentin tubules.

Even though the participants baseline characteristics demonstrated a sample homogeneity, and the non-carious cervical lesions (NCCL) depth was up to 2mm aiming to control this factor, the different lesions depths of the sensitive NCCL could be considered a limitation of the current study since deeper lesions could turn the DH regression a more complex process [49]. Even if there is no universal conduct based on the NCCL depth, some authors report that with increasing depth the plaque accumulation and its irritants effects are more pronounce demanding a restorative approach [30,50]. On the other hand, the etiology of the NCCL does not seem an interference aspect in the treatment response, since the NCCL has been defined as a multi-factorial condition [31,51].

The DHEQ presents excellent psychometric properties with an adequate reliability (General correlation > 0.4; Cronbach’s alpha = 0.86) and validity (r = 0.92), properly assessing functional and personal changes in patients with DH [25,26]. After one-month follow-up CPP-ACPF+PBM participants presented “lower values” in the overall result of the DHEQ suggesting an improvement in the HRQL in the intragroup and intergroup evaluation, and reveals the effectiveness of desensitizing treatment, rejecting H01.

The DHEQ and VAS results confirmed the impact of the desensitizing treatments on DH and the HRQL of the participants of this investigation. CPP-ACPF with a PBM synergistic effect was effective in DH treatment and promoted a positive impact on the HRQL of participants. Further studies should be performed to evaluate the protocols of combination therapies with longer treatment periods.

## Conclusion

Within the limitations of this RCT, it was concluded that the association of CPP-ACPF with PBM was effective in reducing DH after one-month follow-up and promoted a positive impact on the HRQL of the participants of this study.

## Supporting information

S1 Checklist 2010-CONSORT Checklist. (DOCX)

S1 Protocol Copy of trial protocol as approved by the ethics committee. (DOCX)
