# Supplementary material for: Effect of the casein phosphopeptide-amorphous calcium phosphate fluoride (CPP-ACPF) and photobiomodulation (PBM) on dental hypersensitivity: A randomized controlled clinical trial
Source: PLoS One. 2019 Dec 2;14(12):e0225501. doi: 10.1371/journal.pone.0225501 (PMC6886796; doi:10.1371/journal.pone.0225501)
Supplement: S1 Protocol — (DOCX) [file pone.0225501.s002.docx]

**FEDERAL UNIVERSITY OF PARÁ**

**POST-GRADUATE PROGRAM**

**Research Department**

**Effect of CPP-ACPF and low-level laser on symptomatology and quality of life of patients with dentin hypersensitivity: Clinical, randomized, double-blind, placebo-controlled study.**

🖂 **Augusto Corrêa St, 1 (University) - 66075-900 Belém PA – Brazil**

🕿 (091) 3201 7971 - Fax: (091) 3201 7657

**RESEARCH PROJECT**

**1 - IDENTIFICAÇÃO DO PROJETO**

Project’s title: Effect of CPP-ACPF and low-level laser on symptomatology and quality of life of patients with dentin hypersensitivity: Clinical, randomized, double-blind, placebo-controlled study.

Big knowledge area: Health Science

Knowledge area: Dentistry (4.02.00.00.0).

Subarea: Operative dentistry (4.02.04.000).

INSTITUTION: Federal University of Pará

CENTER/DEPARTMENT: Center for Health Sciences / Faculty of Dentistry

EXECUTING UNIT: Faculty of Dentistry

ADDRESS: Av. Augusto Corrêa, nº 01 - Cidade Universitária José da Silveira Netto

| MUNICÍPIO  Belém | CEP  66640480 | U.F.  PA | TEL/FAX  91-32017494 | E-MAIL  [cecymsilva@gmail.com](mailto:cecymsilva@gmail.com) |
| --- | --- | --- | --- | --- |

PROJECT COORDINATOR: CECY MARTINS SILVA

DEPARTMENT: FACULTY OF ODONTOLOGY

OTHER PARTICIPATING INSTITUTIONS

**2 – PROJECT TEAM MEMBERS**

| **Registration** | **Full Name** | **Type*** | **Highest degree** | **Department** | **Function in project**** | **Project load** |
| --- | --- | --- | --- | --- | --- | --- |
| 0327584 | Cecy Martins Silva | PE | PhD | Dentistry Faculty | CD | 10h |
| 0327879 | Eliane Bemerguy Alves | PE | PhD | Dentistry Faculty | CL | 5h |
|  | Mariángela Ivette Guanipa Ortiz | CL | MSc student | Dentistry Faculty | CL | 5h |
|  | Cristiane de Melo Alencar | CL | MSc student | Dentistry Faculty | CL | 5h |
|  | Brennda Lucy Freitas de Paula | CL | MSc | Dentistry Faculty | CL | 5h |
|  | Danielle da Silva Pompeu | CL | Graduate student | Dentistry Faculty | CL | 5h |
|  | Antônia Patrícia de Oliveira Barros | CL | Graduate student | Dentistry Faculty | CL | 5h |
|  |  |  |  |  |  |  |

* TA: Technical administrator

** CD: Coordination

PV: Professor visiting

CL: Collaborator

PE: Permanent Professor

CS: Consultor

PP: Participating Professor

PPE: Professor with external participation

TE: External technical administrator

PB: Professor with funding from a governmental agency

**RESEARCH PROJECT**

**3 – INTRODUCTION**

Dentin hypersensitivity (HD) is defined as: "Acute and short-lived pain from exposed dentin on a thermal, evaporative, tactile, osmotic or chemical stimulus, and which cannot be attributed to any other form of dental defect or pathology ". (1,2,3) The reported pain is of short duration and is remission when the stimulant is withdrawn, affecting the patients' quality of life, which can compromise daily activities such as brushing teeth, eating, drinking, and even social interaction. (4,5)

Non-carious cervical lesions (LCNC) are characterized by the irreversible loss of cervical tooth structure at the cement-enamel junction level (JCE). Among the etiological factors of these lesions we have stress, biocorrosion and friction, and according to the factor that predominates in their development the LCNC are divided into: Abfraction that results mainly from the occlusal trauma that produces tensions in the JCE, generating microfractures in the enamel and dentin exposure. Dental brushing, associated with biocorrosion due to changes in diet, produces tooth wear. (2,6) Because of the irreversible loss of cervical tooth structure that originates, any LCNC could be linked to the development of HD. (7)

Loss of gingival-tooth bonding or gingival recession is another factor related to the exposure of the dentinal tubules at the cemento-enamel junction. Since the rate of cement resorption is greater than that of dentin, a root exposure increases, increasing the probability of opening the dentinal tubules to the oral medium, this whole cycle results in HD. (8)

The hydrodynamic theory of Brännström is accepted to describe the origin of HD, which is initiated by thermal, mechanical or chemical stimuli acting on the exposed dentin tubules, altering the movement of the fluid inside them and stimulating the response of the fibers A-β and A-δ (which are surrounding the odontoblasts), which react in the form of pain. (1,2) According to this theory, the best treatment for sensitivity is the reduction of fluid movement within the tubules by obstructing them, or by limiting the nerve response. (9)

The ideal desensitizing material should exhibit rapid action, long-term effect, be biocompatible, non-irritating to the pulp, painless, easy to apply, and not cause tooth color change. (10,11) The fluorinated amorphous calcium casein-phosphate (CPP-ACPF) phosphopeptide may be useful in reducing HD because of its ability to incorporate into the surface of the enamel and dentin, acting as a reservoir of calcium and phosphate, occluding the dentinal tubules, avoiding the movement of liquid inside them, preventing the stimulation of the nerve fibers, and therefore the HD. (12)

Casein phosphopeptides (CPP) originate by enzymatic hydrolysis of casein. CPP has multiple phosphoserine residues, which interact and bind to calcium and amorphous phosphate (ACP) ions stabilizing them in an aqueous solution, forming the CPP-ACP complex. When fluoride is added, it becomes phosphopeptide of casein-fluorinated amorphous calcium phosphate (CPP-ACPF), which is more effective than CPP-ACP to inhibit enamel demineralization and facilitate remineralization of enamel early lesions by the availability of high concentrations of calcium and phosphate. (13,14)

Low power laser therapy (TLBP) involves the noninvasive application of red light (600-700 nm) and near infrared (700-950 nm), with a power density (irradiance) between 01 mW and 5 m W / cm2, (15) being increasingly employed in medicine and dentistry due to its analgesic, anti-inflammatory and biostimulating effects. It acts by inducing the production of mitochondrial adenosine triphosphate (ATP), increasing the presence of β-endorphin, and by inhibiting the enzyme cyclooxygenase, which transforms arachidonic acid into prostaglandins, attenuating the perception of painful stimuli in dentin tissues. (16) TLBP also reduces dentin sensitivity by means of photobiomodulation of the dental pulp, generating an increase in the cellular activity rate of odontoblasts, increasing its production of tertiary dentin, thus obliterating the dentinal tubules. (17,18)

**4 - JUSTIFICATIVE**

The prevalence of HD ranges from 8% to 74% according to the population evaluated and the technique used for its diagnosis. (8, 19, 20, 21) But almost all studies agree that the predominant location is in the canines and first premolars, followed by incisors and second premolars. (22) An investigation in the Northwest of the United States evaluated 787 adult patients and found a prevalence of 12.3% in the 18-44 age group, predominating in women, and associated with periodontal disease, gingival recession, or erosive exposure. (23) Of the 300 Brazilian patients, 46% had HD, with a mean age of 40 years, where toothbrushing four times a day and with excessive force, bruxism and / or gastroesophageal reflux were associated factors. (24)

Now, in countries such as Canada, with a history of long-term preventive dentistry, consequently there is a large number of elderly teeth, and according to the Canadian Advisory Board (1), this makes the prevalence rate of HD in this country from 25 to 30% of the population and can not be related to age. However, the same situation can not be extrapolated worldwide, it is estimated that a quarter of its population suffers from HD, although this is not a lethal disease, but it is clinically relevant since it severely compromises the quality of life of its patients . Therefore, the evaluation of effective and long-lasting desensitizing treatments is necessary to address the consequences of HD. (25)

Dentin and pulp have the same ectomesenchymal embryological origin and are closely related, that is, the physiological and / or pathological reactions in one tissue will affect the other. (26, 27) Dentin has a large amount of dentin tubules, and each one is occupied by an odontoblastic process and the surrounding dentin, the latter being responsible for communicating the pulp to the outermost regions of the dentin. When the dentine is exposed by loss of enamel or cementum suprajacent, changes in the medium may affect the histophysiology of its dentin fluid, and consequently the nerve fibers of the dentin-pulp complex generating pain, which may be reduced by occlusion of the tubules exposed. (27, 28)

Absi et al. (29) reported the correlation between HD and dentin tubule density. By means of scanning electron microscopy (SEM), they determined that, in sensitive teeth, the number of open tubules and their amplitude were respectively eight and two times higher when compared to teeth without HD. In physics, the flow of the liquid is proportional to the fourth power of the radius, indicating that twice the diameter in the teeth with HD generates a 16 times greater flow than in the healthy teeth. (2,22)

For the development of HD, two events are essential: "lesion location" - dentin is exposed by removal of enamel or dental cementum, and "initiation of injury" - by opening the dentinal tubules to the buccal environment. (1, 3, 22) Since treatments that occlude the dentinal tubules are ideal for reducing HD, however, their efficacy will depend on their resistance to the various challenges of the oral environment. (3)

CPP-ACP is a milk protein derived complex marketed as an additional source of calcium and phosphate ions. When fluoride is added to the CPP-ACP, the deposition of calcium, phosphate and fluoride ions on the tooth, the apatite crystals reorganize and the surface remineralize, recovering the properties of the dental structure. (30, 31)

Due to the low solubility of calcium phosphate ions (PCA) and their poor adhesion to the dental surface, a stabilizing agent is required, such as casein phosphate (CPP) whose multiple phosphorylated serine groups solubilize and stabilize the ACP , maintaining high concentration gradients of calcium and phosphate ions in a demineralised lesion, promoting high rates of enamel remineralization. (32, 33)

Similarly, fluorine is a remineralizing agent by being converted to fluorapatite (FA). However, to form one unit of FA, ten calcium ions and six phosphate ions are required for every two fluorine ions (Ca10 (PO4) 6 F2), and consequently the availability of calcium and phosphate ions restricts the formation of the remineralizing network by the fluorine. CPP-ACPF has a synergistic effect on dental remineralization, since CPP-ACP increases the concentration of calcium and bioavailable phosphate ions, thereby increasing the incorporation of fluoride ions into the dental biofilm, as well as, in the subsurface lesions of enamel in the form of FA. (13, 34, 35)

When CPP-ACP and a placebo gel were evaluated, under the effect of decreasing HD during 6 months, CPP-ACP demonstrated a rapid and stable reduction of HD during follow-up, whereas placebo generated an improvement moderate, but this was not statistically significant. (36) This is in agreement with Tung et. Al 1992, which demonstrated that, at high concentrations, CPP-ACP precipitates amorphous calcium phosphate, which, being the main inorganic component of the tooth, decreases dentin permeability by 85%, acting as an excellent tubular obliterator. (36)

On the other hand, TLBP has been applied to promote wound healing, reduce inflammation, and alleviate chronic and acute pain. This latter effect is related to the suppression of the activity of the substance P and bradykinin, serotonin release, improved circulation reduction in the production of acid metabolites that stimulate pain receptors, and deceleration of the action potential velocity, inhibiting the electrical conduction of Fibers C and Aδ, and modulating the pain threshold. However, the therapeutic effects of TLBP are subject to the application of certain wavelengths, laser intensity, location and optical properties of treated tissues. (15)

The diode laser of arsenic gallium and aluminum (AsGaAl), with a wavelength of 810 nm, increases the action potential of nerve cells, thus limiting the transmission of painful stimuli, but without heating the dental surface to the degree of generating modifications in the same. (37) With other wavelengths, the increase in energy in the dental surface leads to dentin fusion, and reduction of the diameter or obliteration of the dentinal tubules. (9, 38) A recent systematic review has shown that AsGaAl laser is more effective in reducing HDD in the long term than certain desensitizing agents without causing pulpal damage, allergy, or obvious clinical complications. (21)

However, although there are multiple treatments to reduce HD, none of them are considered a "gold standard", since the most commonly used desensitizing agents are effective in the short term, with an unstable response rate in more extended times (39,40) the need for clinical trials to evaluate the effectiveness of desensitizing treatments over time and to clarify the methodology and ideal product for the treatment of this disease due to the impact on the quality of life of its patients.

CPP-ACPF and TLBP present the ability to reduce dentin sensitivity by obliterating the dentinal tubules, and their association could generate a synergistic effect, being a more effective way of treating HD, justifying the purpose of this study.

**RESEARCH PROJECT**

**5 – AIM**

The aim of this study was to evaluate the effect of the fluorinated amorphous calcium phosphate casein-phosphorus (CPP-ACPF) associated with low-power laser therapy (TLBP) on the symptomatology and impact of the quality of life of patients with dentin hypersensitivity ( HD) by means of a randomized, parallel, placebo-controlled clinical trial. The null hypothesis tested in the present study will be:

H0 - there will be no difference in the manifestation of dentin hypersensitivity between the groups treated with TLBP, CPP-ACPF or placebo, associated or not, during one month of evaluation, and will not influence the participants' quality of life.

**6 – METHODOLOGY**

## **6.1. Ethical aspects**

This research project will follow the recommendations of the CONSORT (Consolidated Standards of Reporting Trials), (41). The research participants will be duly informed and informed about the risks, methods and objectives of this project. It is necessary to sign the informed consent form (TCLE) in accordance with the Helsinki declaration before starting treatment.

The information to be collected was exclusively scientific and the identity of the participants was preserved throughout the study. Participation in the study could be canceled and the consent term withdrawn at any time from the research, ensuring confidentiality of the participant even in case of abandonment of the treatment.

**6.2. Sample selection**

Students and employees of the Federal University of Pará, Belém, Pará, Brazil, between 18 and 50 years old, of both sexes, will be evaluated and recruited at the Faculty of Dentistry of the Federal University of Pará, Belém, Pará, Brazil. The inclusion and exclusion criteria are described in Table 01.

Two calibrated dentists evaluated potential candidates. Firstly, using a tactile stimulus: by sliding an exploratory catheter 5 (Exploratory Probe, Ice, Sao Paulo, Brazil) horizontally in the mesio-distal direction and vertically in the cervico-incisal direction of the cervical third of the selected teeth, with a light force. After stimulation, patients will be asked to point the 10-cm-long analogue visual scale (VAS) at the number that describes their perception of pain (0 = absent pain and 10 = extreme pain). After five minutes, the evaluator will apply an air stimulus: throwing a 3-second air jet of a triple syringe (Triple Syringe, Dabi Atlante, São Paulo, Brazil) with ± 40psi of pressure, directed perpendicular to the surface of the lesion and 0.5 cm apart. Protecting neighboring teeth through cotton rolls or by sealing with operator's fingers. Participants will again be asked to assign the corresponding pain score on the EVA scale

He commented that the main inclusion criterion will be the presence of two to four teeth with HD, in order to obtain groups with similar sample sizes (n) and facilitate the statistical comparison. In case the patient has more than four teeth with HD, for statistical analysis will be considered those with greater sensitivity according to EVA.

**Table 01: Selection criteria.**

| - INCLUSION CRITERIA | | ^x^ EXCLUSION CRITERIA |
| --- | --- | --- |
| - Presence of dentin exposure in anterior and / or posterior teeth - presence of two to four teeth with hypersensitive dentin, with a response ≥04 on the 10 cm long visual analogue scale (VAS). - Non-carious shallow lesion up to 2 mm deep, according to the Smith & Knight index (measured with a millimeter probe). (42) - Class I gingival recession according to Miller's classification. (43) | - Milk protein allergies; - Systemic diseases; - Presence of pulpitis; - Carious lesions; - Presence of parafunctional habits. - Presence of restorations in the cervical area, or defective in the tooth; - Presence of periodontal disease; - Presence of cracks or fractures in the enamel; - Patients in fixed orthodontic treatment; - Pregnant or lactating women; - Medication with analgesics; - Desensitizing treatment during the three months prior to the research recruitment date. | |

**6.3. Sample Calculation**

To determine the sample size of the study, data from a pilot study that followed the methodology proposed in this research project was used, with four participants, three (75% of the sample) being female and one (25% of the sample) of the sample. male, with mean age of 31 years and standard deviation of ± 1.91. In the pilot study, 16 teeth were randomly assigned to one of the four groups with the Research Randomizer Calculator (www.socialpsychology.org/randomizer.htm).

The VAS was used to measure sensitivity, considering a clinically relevant difference of three units between the initial and final VAS scores, according to a previous study (20). The G Power® program (Heinrich-Heine-Universität Düsseldorf, Germany) was used for mixed ANOVA, considering a statistical power of 80%, α error of 5% and prediction of sample loss at the end of the study of 20%. The resulting sample for this study was 80 teeth, 20 teeth per group.


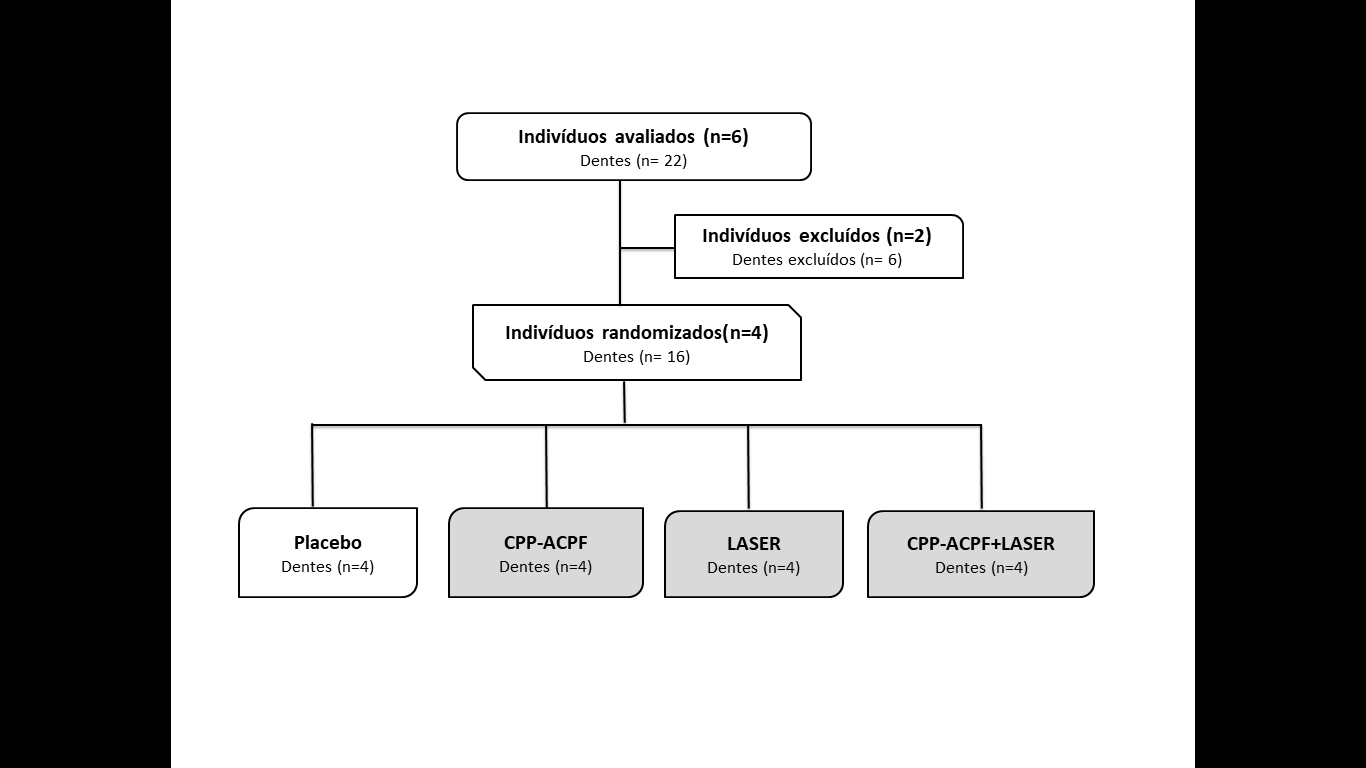


**Flowchart 01. Recruitment and allocation scheme performed in the pilot study**

**6.4. Study design**

After randomization of the sample, the teeth (at least 20 for each group) will be allocated to one of the four assessment groups according to the scheme below:
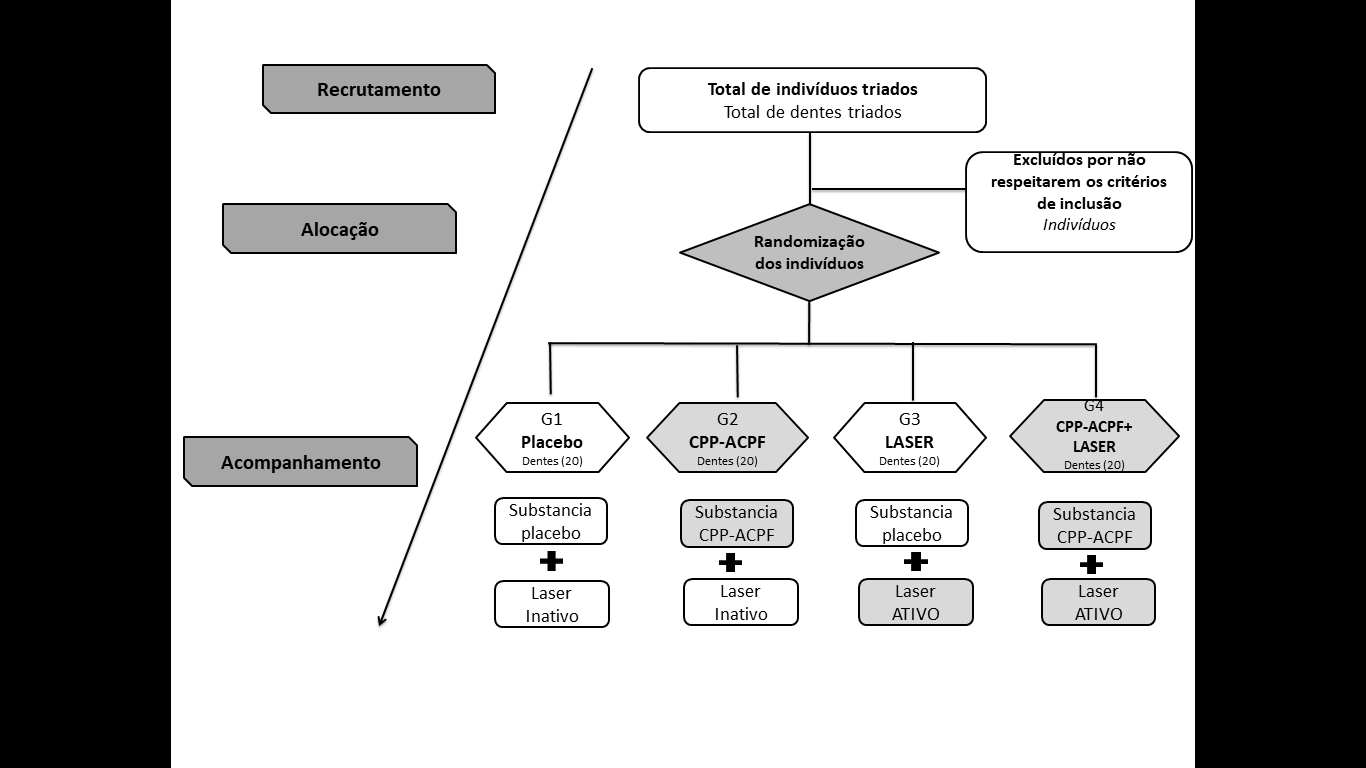


**Flowchart 02. Experimental design of the study**

**Table 2.** Division of desensitizing groups and treatments.

| **GROUPS** | **DENSENSIBILIZING TREATMENT** |
| --- | --- |
| **Gplacebo** | Water-soluble placebo gel (K-Y®, Johnson & Johnson, SP, Brazil) with no active agent. |
| **GCPP-ACPF** | MI Paste Plus ™ (Recaldent ™, GC America, United States) |
| **Glaser** | Application of Laser - Photon lase III DMC |
| **GLaser +CPP-ACPF** | Application of Laser - Photon lase III DMC + MI Paste Plus ™ (Recaldent ™, GC America, United States) |

In order to standardize the initial condition, participants will be submitted to prophylaxis with rubber cup (Unid Microdont, São Paulo, SP, Brazil) and pumice stone (Maquira, Maringá, PR, Brazil). In order to mitigate possible interferences in the follow-up of the study, they received a dental hygiene kit composed of a soft bristle toothbrush (Top Plus, Condor, São Bento do Sul, SC, Brazil) and a dentifrice without desensitizing action. contains fluoride (EVEN Baby, Raimundo da Fonte Group, Paulista, PE, Brazil), with guidance on how to brush and with a frequency of at least three times a day.

**6.5. Randomization**

The participants, with at least two teeth with HD equal or superior to four in the EVA, will be allocated through a block randomization aiming to balance the sample number in the amount of existing arms. For each block a total of four participants were defined, and after the formation of the block, a numerical draw among the participants was carried out and, subsequently, each 01 volunteer was allocated to compose one of the four groups under different arrangements (A4,1 , A3.1, A2.1 and 1) (Figure 01). The total number of blocks will be ± 10.

Allocation secrecy will be maintained throughout the sampling composition process. One of the project's collaborators will be responsible for the formation of the block, numerical draw within the block and allocation in the groups. The numerical draw will be done using numbered and coded papers, of which the participants, the principal investigator, the clinical operator and the evaluator will not be aware of the group to which the participant will be allocated.


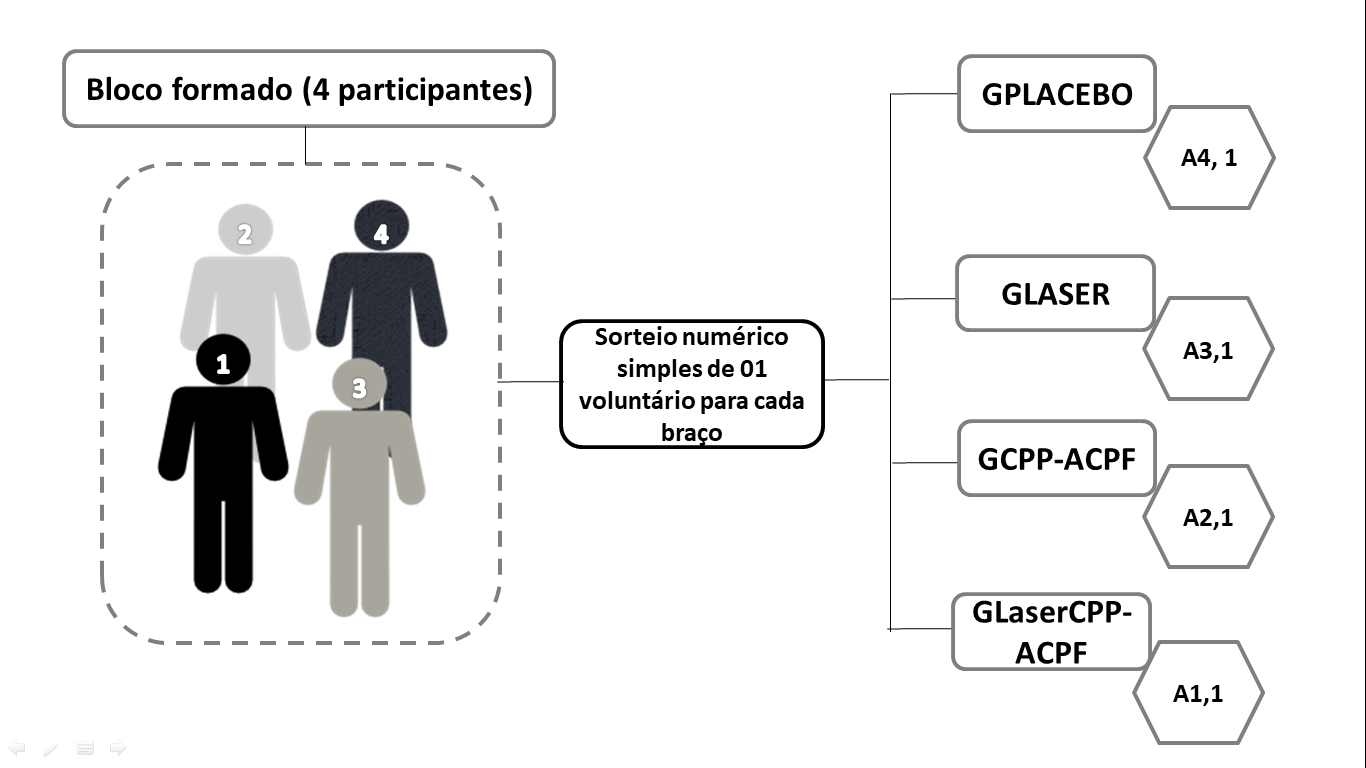


**Figure 01. Randomization scheme within a block and possible arrangements for the allocation in the arms**

**6.6. Blinding**

This study will be double-blind. The research will rely on only one operator, who has performed the experimental steps of the research. And it will involve only one evaluator to check the pain sensitivity, who will not participate in the randomization process, nor will he be aware in which group the patient will be inserted. Participants evaluated in the experiment will also not be aware of the treatment to which they will be submitted. The desensitizing gel and the placebo gel will be placed in equal containers to avoid identification by the patients of the applied product.

**6.7. Clinical Protocol:**

Frequency of treatment: Three sessions of desensitizing treatment will be done, with a 24-hour interval between sessions.

**6.7.1. CPP-ACPF and Placebo**

The subjects of G. CPP-ACPF will receive the application of the MI Paste Plus ™ gel (Recaldent ™, GC América, United States) on the vestibular surfaces of hypersensitive teeth with the aid of a Microbrush applicator (Microbrush, 3M ESPE, São Paulo, Brazil) and was maintained undisturbed for 5 min. A rubber cup mounted on a low speed handpiece will then be used to scrub the desensitizing gel for 20 seconds on each tooth as per the manufacturer's specification (http://www.mi-paste.com/apply.php) . After application of the CPP-ACPF dentifrice, the TLBP will be simulated with the positioning in two points: one in the center of the middle third and the other in the cervical third, without laser light emission, and the sound of the equipment will be mimicked with the Beep application (Foncannon Inc, Google, © 2018).

The placebo group will receive the same protocol as the CPP-ACPF group, but the gel used will be a water-soluble base with no active ingredient (K-Y®, Johnson & Johnson, SP, Brazil).

**6.7.2. Laser therapy and laser therapy associated with CPP-ACPF**

In the laser group, the laser will be applied using an infrared light spectrum with wavelength of 808 nm with its active medium AsGaAl, positioning the tip in two points, one positioned in the center of the middle third and the other in the cervical third. It will be used at each point 60 J / cm², for 16 seconds, using Photon Laser III Therapeutic (DMC Equipamentos, São Carlos, SP, Brazil, Ltda). Before application of the laser, the placebo gel will be applied as previously described.

In the laser + CPP-ACPF group, CPP-ACPF will be applied following the same protocol of G. CPP-ACPF, and then the laser will be applied in the same way as in the laser group.

## **6.8. Assessment of pain sensitivity**

For the evaluation of pain sensitivity, the visual analogue scale (EVA) of 10 cm in length will be used, where 0-pain is absent and 10-pain is extreme. Requesting the participant to signal the number compatible with the pain sensation after application of the stimuli, evaporative (triple syringe) and tactile (exploratory probe). Sensitivity assessment will be done in five moments: Immediately before starting treatment, after the first session, after the second session, after the third session and after one month of treatment.

### **6.8.1. Evaporative Stimulation**

### The evaporative stimulus will consist of the application of a 40 psi air pressure of the triple syringe for 03 seconds, being directed perpendicular to the vestibular face of the tooth at a distance of 0.5 cm, the pain response will be measured by the participant according to the EVA . (44)

### **6.8.2. Tactile Stimulus**

### The tactile stimulus will be realized from the contact of the exploratory probe in the form of cross. The contact will be performed vertically in the cervico-incisal direction and horizontally in the mesio-distal direction, in the cervical region of the hypersensitive teeth. And the HD degree will be recorded with the help of EVA. (44)

### **6.8.3. Dentin Hypersensitivity Experience Questionnaire (QEHD).**

### Participants will be given a questionnaire prior to the start of treatment, and after one month thereafter, to assess the impact of desensitizing treatment on their quality of life. The questionnaire is based on the model described by Boiko et. Al. (45) according to the Portuguese adaptation of Douglas-De-Oliveira et. Al. (46). Also, dietary habits, systemic diseases and parafunctional habits of the participants will be recorded through a questionnaire, in order to analyze through a regression analysis the possible interaction of these factors with the HD reported throughout the study.

## **6.9. Statistical analysis**

### The values ​​corresponding to the sensitivity reported by the participants were tabulated in an Excel spreadsheet (Microsoft Windows, 2010) and analyzed with the IBM SPSS Statistics for Windows 22.0 program (IBM Corp., New York, United States). Considering the parametric data of this study, if it has normal distribution of the same to the intergroup analysis will be done with mixed ANOVA, and the specific intergroup comparison will be done with the Tukey post-hoc test. If the data are abnormal distribution the intragroup comparison will be performed from the Friedman test and the intergroup by the Wilcoxon test. A significance level of 5% will be set for all analyzes to be performed.

### To analyze non-parametric QEHD data the Wilcoxon test will be used.

**7 - GOALS**

### The present project aims to be developed within the timeframe set forth in the schedule and to start after approval by the bioethics committee. The bibliographic review will be done from September 2017 until December 2018, the work will be submitted to the bioethics committee and inserted in an international clinical records site between August and September 2017, followed by the screening period from October until November 2017. Upon completion of the screening, the randomized clinical trial will be conducted from November 2017 through September 2018. After data collection is completed, statistical analysis of the results will be done from October 2018 through November 2018. The final report will be built and submitted by December 2018 and later the scientific paper will be written for publication.

**8 - BIBLIOGRAPHY**

1. Canadian Advisory Board on Dentin Hypersensitivity. Consensus-based recommendations for the diagnosis and management of dentin hypersensitivity. J Can Dent Assoc. (2003); 69: 221-226.
2. West N.; Sanz M.; Lussi A.; Seong J.; Hellwig E. Dentin hypersensitivity: pain mechanisms and aetiology of exposed cervical dentin. Clin Oral Invest. (2013); 17: 9-19.
3. West N.; Seong J.; Davies M. Management of dentine hypersensitivity: efﬁcacy of professionally and self-administered agents. J Clin Periodontol. (2015); 42: 256–302.
4. Douglas-de-Oliveira D.; Pereira G.; Oliveira J.; Castro C.; Oliveira F.; Miranda L. Effect of dentin hypersensitivity treatment on oral health related quality of life-A systematic review and meta-analysis. J of Dent. (2017); In press, corrected proof.
5. Torwane N.; Hongal S.; Goel P.; Chandrashekhar B.; Jain M.; Saxena.; Gouraha A.; Yadav S. Effect of Two Desensitizing Agents in Reducing Dentin Hypersensitivity: An in-vivo Comparative Clinical Trial. J Clin Diagn Res. (2013); 7: 2042-2046.
6. Gojkov-Vukelic M.; Hadzic S.; Zukanovic A.; Pasic E.; Pavlic V. Application of Diode Laser in the Treatment of Dentine Hypersensitivity. Med Arc. (2016); 70: 466–469.
7. Grippo J., Simring M., Coleman T. Abfraction, abrasion, biocorrosion, and the enigma of noncarious cervical lesions: a 20-year perspective. J Esthet Restor Dent. (2012); 24:10-23.
8. Mahajan G.; Kaur H.; Gautam A. Prevalence of buccal cervical dentine hypersensitivity and related risk factors - A cross-sectional study. Int Dent Med J Adv Res (2017); 3:1-5.
9. Sgolastra F.; Petrucci A.; Severino M.; Gatto R.; Monaco A. Lasers for the Treatment of Dentin Hypersensitivity: A Meta-analysis. J Dent Res. (2013); 92: 492-499.
10. Grossman L. A systematic method for the treatment of hypersensitive dentine. J Am Dent Assoc. (1935); 22: 592–598.
11. Lopes A.; De Paula C.; Correa A. Clinical evaluation of low-power laser and a desensitizing agent on dentin hypersensitivity. Lasers Med Sci. (2015); 30: 823–829.
12. Kowalczyk A.; Botuliński B.; Jaworska M.; Kierklo A.; Pawińska M.; Dąbrowska E. Evaluation of the product based on Recaldent TM technology in the treatment of dentin hypersensitivity. Adv Med Sci. (2006); 51: 40-42.
13. Huq N, Myroforidis H, Cross K, Stanton D, Veith P, Ward B, Reynolds E. The Interactions of CPP-ACP with Saliva. Int J Mol Sci. (2016); 9: 1-7.
14. Cochrane N.; Reynolds E. Calcium phosphopeptides - mechanisms of action and evidence for clinical efficacy. Adv Dent Res. (2012); 24: 41-47.
15. Pandeshwar P.; Roa M.; Das R.; Shastry S.; Kaul R.; Srinivasreddy M. Photobiomodulation in oral medicine: a review. J Investig Clin Dent. (2016); 7: 114-126.
16. Pandey R.; Koppolu P.; Kalakonda B.; Lakshmi B.; Mishra A.; Reddy P.; Bollepalli A. Treatment of dentinal hypersensitivity using low-level laser therapy and 5% potassium nitrate: A randomized, controlled, three arm parallel clinical study. Int J Appl Basic Med Res. (2017); 7: 63-66.
17. Romeo U.; Russo C.; Palaia G.; Tenore G.; Del Vecchio A. Treatment of Dentine Hypersensitivity by Diode Laser: A Clinical Study. Int J of Dent. (2012); 2012: 1-8.
18. Lopes O.; De Paula E.; Correa A. Evaluation of different treatment protocols for dentin hypersensitivity: an 18-month randomized clinical trial. Laser Med Sci. (2017); 32: 1023-1030.
19. Mantzourani M.; Sharma D. Dentine sensitivity: past, present and future. J of Dent. (2013); 41: 3–17.
20. Schmidlin P.; Sahrmann P. Current management of dentin hypersensitivity. Clin Oral Invest. (2013); 17: 55–59.
21. He S.; Wang Y.; Li X.; Hu D. Effectiveness of laser therapy and topical desensitising agents in treating dentine hypersensitivity: a systematic review. Journal of Oral Rehabilitation. (2011); 38: 348–358.
22. Addy M. Dentine hypersensitivity: New perspectives on an old problem. Int Dent J. (2002); 52: 367-375.
23. Cunha-Cruz J.; Wataha J.; Heaton L.; Rothen M.; Sobieraj M.; Scott.; Berg J. The prevalence of dentin hypersensitivity in general dental practices in the northwest United States. J Am Dent Assoc. (2013); 144: 288-296.
24. Scaramucci T.; de Almeida Anfe T.; da Silva Ferreira S.; Frias A.; Sobral M. Investigation of the prevalence, clinical features, and risk factors of dentin hypersensitivity in a selected Brazilian population. Clin Oral Investig. (2014); 18: 651–657.
25. Splieth C.; Tachou A. Epidemiology of dentin hypersensitivity. Clin Oral Investig. (2013); 17: 3-8.
26. Davari A.; Ataei E.; Assarzadeh H. Dentin Hypersensitivity: Etiology, Diagnosis and Treatment: A Literature Review. J Dent. (2013); 14: 136–145.
27. Miglani S.; Aggarwal V.; Ahuja B. Dentin hypersensitivity: Recent trends in management. J Conserv Dent. (2010); 13: 218–224.
28. Bae J.; Kim Y.; Myung S. Desensitizing toothpaste versus placebo for dentin hypersensitivity: a systematic review and meta-analysis. J Clin Periodontol. (2015); 42: 131-141.
29. Absi E.; Addy M.; Adams D. Dentine hypersensitivity. A study of the patency of dentinal tubules in sensitive and non-sensitive cervical dentine. J Clin Periodontol. (1987); 14: 280-284.
30. Zenouz A.; Ezoji F.; Enderami S.; Khafri S. Effect of Fluoride, Casein Phosphopeptide–Amorphous Calcium Phosphate and Casein Phosphopeptide–Amorphous Calcium Phosphate Fluoride on Enamel Surface Microhardness After Microabrasion: An in Vitro Study. J Dent (2015); 12: 705–711.
31. Alexandrino L.; Alencar C.; Silveira A.; Alves E.; Silva C. Randomized clinical trial of the effect of NovaMin and CPP-ACPF in combination with dental bleaching. J App Or Sci (2017); 25: 335-340.
32. Nongonierma A.; Fitzgerald R. Biofunctional properties of caseinophosphopeptides in the oral cavity. Caries Res. (2012); 46: 234-267.
33. Penumatsa N.; Kaminedi R.;, Baroudi K.; Barakath O. Evaluation of remineralization capacity of casein phosphopeptide-amorphous calcium phosphate on the carbamide peroxide treated enamel. Dent Sci. (2015); 7: 583-586.
34. Soares R.; De Ataide I.; Fernandes M.; Lambor R. Assessment of Enamel Remineralisation After Treatment with Four Different Remineralising Agents: A Scanning Electron Microscopy (SEM) Study. J Clin Diagn Res. (2017); 11: 136-141.
35. Reynolds E.; Cai F.; Cochrane N.; Shen P.; Walker G.; Morgan M.; Reynolds C. Fluoride and Casein Phosphopeptide-Amorphous Calcium Phosphate. J Dent Res. (2008); 87: 344-348.
36. Geiger S.; Matalon S.; Blasbalg J.; Tung M.; Eichmiller F. The clinical effect of amorphous calcium phosphate (ACP) on root surface hypersensitivity. Oper Dent. (2003); 28: 496-500.
37. Bal M.; Keskiner I.; Sezer U.; Açıkel C.; Saygun I. Comparison of low level laser and arginine-calcium carbonate alone or combination in the treatment of dentin hypersensitivity: a randomized split-mouth clinical study. Photomed Laser Surg. (2015); 33: 200-205.
38. Liu Y.; Gao J.; Gao Y.; Xu S.; Zhan X.; Wu B. In Vitro Study of Dentin Hypersensitivity Treated by 980-nm Diode Laser. J Lasers Med Sci. (2013); 4: 111-119.
39. García-Delaney C.; Abad-Sánchez D.; Arnabat-Domínguez J.; Valmaseda-Castellón E.; Gay-Escoda C. Evaluation of the effectiveness of the photobiomodulation in the treatment of dentin hypersensitivity after basic therapy. A randomized clinical trial. J Clin Exp Dent. (2017); 9: 694-702.
40. Wang L.; Magalhães A.; Francisconi-Dos-Rios L.; Calabria M.; Araújo D.; Buzalaf M.; Lauris J.; Pereira J. Treatment of Dentin Hypersensitivity Using Nano-Hydroxyapatite Pastes: A Randomized Three-Month Clinical Trial. Oper Dent. (2016); 41: 93-101.
41. Pannuti C.; Rosa F.; Guglielmetti M.; Moreira R. Avaliação Crítica de Ensaios Clínicos. Rev Odontol Bras Central. 2011; 52: 20.
42. Smith BG.; Knight JK. An index for measuring the wear of teeth. Br Dent J. 1984; 156:435-8.
43. Miller PD Jr. A classification of marginal tissue recession. Int J Periodontics Restorative Dent. 1985; 5:8-13.
44. Holland GR.; Narhi MN.; Addy M.; Gangarosa L.; Orchardson R. Guidelines for the design and conduct of clinical trials on dentine hypersensitivity. J Clin Periodontol. 1997; 24: 808-13.
45. Boiko O.; Baker S.; Gibson B.; Locker D.; Sufi F.; Barlow A.; Robinson P. Construction and validation of the quality of life measure for dentine hypersensitivity (DHEQ). J Clin Periodontol. (2010); 37: 973–980.
46. Douglas-De-Oliveira DW.; Lages FS.; Paiva SM.; Cromley JG.; Robinson PG.; Cota LOM. Cross-cultural adaptation of the Brazilian version of the Dentine Hypersensitivity Experience Questionnaire (DHEQ-15). Braz Oral Res. (2018); 32:e37.

**7 – SCHEDULE OF ACTIVITIES**

| **ACTIVITIES** | **2017** | | | | | | **2018** | | | | | | | | | | | | **2019** |
| --- | --- | --- | --- | --- | --- | --- | --- | --- | --- | --- | --- | --- | --- | --- | --- | --- | --- | --- | --- |
|  | **Jul** | **Ag** | **Set** | **Ot** | **Nov** | **Dez** | **Jan** | **Fev** | **Marc** | **Abri** | **Maio** | **Jun** | **Jul** | **Ag** | **Set** | **Out** | **Nov** | **Dez** | **Jan** |
| Approval on ethics committee and registration on clinical research site |  |  | x | x |  |  |  |  |  |  |  |  |  |  |  |  |  |  |  |
| Literature review |  |  | x | X | x | x | x |  | x | x | x | x | x | x | x | x | x | x |  |
| Qualification exam |  |  |  |  |  |  |  | x |  |  |  |  |  |  |  |  |  |  |  |
| Development of the randomized clinical trial |  |  |  |  | x | x | x |  | x | x | x | x | x | x | x |  |  |  |  |
| Tabulation and statistical analysis of results |  |  |  |  |  |  |  |  |  |  |  |  |  |  |  | x | x |  |  |
| Elaboration and delivery of the final report. |  |  |  |  | x | x | x |  | x | x | x | x | x | x | x | x | x | x |  |
